# Prediction of bone mineral density in CT using deep learning with explainability

**DOI:** 10.3389/fphys.2022.1061911

**Published:** 2023-01-10

**Authors:** Jeong-Woon Kang, Chunsu Park, Dong-Eon Lee, Jae-Heung Yoo, MinWoo Kim

**Affiliations:** ^1^ Department of Information Convergence Engineering, Pusan National University, Yangsan, South Korea; ^2^ Busan Medical Center, Department of Orthopedic Surgery, Busan, South Korea; ^3^ Department of Biomedical Convergence Engineering, Pusan National University, Yangsan, South Korea

**Keywords:** bone mineral density, computational tomography, dual energy X-ray absorptiometry, deep learning, explainable artificial intelligence

## Abstract

Bone mineral density (BMD) is a key feature in diagnosing bone diseases. Although computational tomography (CT) is a common imaging modality, it seldom provides bone mineral density information in a clinic owing to technical difficulties. Thus, a dual-energy X-ray absorptiometry (DXA) is required to measure bone mineral density at the expense of additional radiation exposure. In this study, a deep learning framework was developed to estimate the bone mineral density from an axial cut of the L1 bone on computational tomography. As a result, the correlation coefficient between bone mineral density estimates and dual-energy X-ray absorptiometry bone mineral density was .90. When the samples were categorized into abnormal and normal groups using a standard (T-score =−1.0 ), the maximum F1 score in the diagnostic test was .875. In addition, it was identified using explainable artificial intelligence techniques that the network intensively sees a local area spanning tissues around the vertebral foramen. This method is well suited as an auxiliary tool in clinical practice and as an automatic screener for identifying latent patients in computational tomography databases.

## 1 Introduction

Osteoporosis, which is characterized by reduced bone mineral density (BMD), is one of the primary causes of fractures (NIH Consensus Development Panel on Osteoporosis Prevention, Diagnosis, and Therapy, 2001). Some studies have reported that more than 200 million people currently suffer from osteoporosis worldwide (Cooper, 1999). Because the average life expectancy has grown in recent years and its prevalence is more common in older people, the total proportion will consistently increase (Cooper et al., 1992). Because osteoporosis reduces quality of life and fractures can result in mortality (Cooper et al., 1993), early diagnosis is crucial for preventing the epidemic. To make the best diagnostic or surgical decisions, physicians must have access to quantitative BMD as well as to anatomical bone images.

Computational tomography (CT) is the main tool used to study bone, however it is seldom used for functional imaging or quantitative measurements. Dual-energy X-ray absorptiometry (DXA) has been widely used by clinicians owing to its non-invasiveness and cost efficiency. The World Health Organization (WHO) still encourages DXA for diagnosing osteoporosis by measuring BMD at the femoral neck and lumbar vertebrae, as well as interpreting individual values with settled statistics, such as T-score (World Health Organization, 1994). Recently, dual-energy CT has been used to extend dimensional information and access local changes in BMD, however it is not yet widespread; it often requires a calibration phantom for every examination, and the radiation exposure is relatively high (van Hamersvelt et al., 2017; Mussmann et al., 2018). One concept was to assess BMD using only CT images without DXA. If realized, it will have a clinical impact because 1) anatomical view and quantitative information can be provided at once, 2) people who underwent CT examination even for routine check-up can be automatically screened, 3) a large CT database can be used to screen latent patients, and 4) additional cost and radiation can be reduced.

Many groups have developed methods to implement this concept. Most studies have reported that diagnosis of osteoporosis is possible with CT attenuation in Hounsfield units (HU), despite a single energy band (Pickhardt et al., 2011; Schreiber et al., 2011; Romme et al., 2012; Pickhardt et al., 2013; Hendrickson et al., 2018). Specifically, they selected a region of bone interest, computed mean HU values in the area for every sample, checked the correlation between the HU values and reference DXA T-scores, and determined HU thresholds corresponding to the T-score border between the normal and abnormal values. Their methods were simple to use, however they were limited to binary classification and could not provide numerical BMD estimates to practically support clinicians.

Currently, deep learning (DL) has been widely used to accomplish complex medical imaging tasks because of its strong generation power (Suzuki, 2017). Some groups applied supervised learning techniques to BMD predictions with the guidance of DXA BMD as a label (reference). Hsieh et al. (2021) developed DL models to detect anatomical landmarks from plain radiographs, automatically select regions of interest (ROI) using the landmarks, and predict BMD values for the lumbar spine and hip. Sato et al. (2022) suggested a DL method to predict BMD from larger views using chest X-rays acquired for diverse medical purposes. The studies commonly demonstrated the potential of the DL approaches, but they limited the use of DL in X-rays less informative than CT. Yasaka et al. (2020) first applied a DL model to CT and obtained high correlations between BMD estimates and DXA references. However, the number of training/testing patient samples was small, and the study had few interpretations with respect to the DL results.

In this study, we focused on standard CT images and aimed to predict BMD using our DL frameworks with explainability. Our main contribution is to achieve state-of-the-art predictions in CT and suggest new algorithms to interpret the numerical DL results. Specifically, the study first considered the selection of a region of interest (ROI) for building neural networks because DXA BMD as a reference depends on not only the bone area but also the surrounding tissues (Bolotin, 2004). We segmented the lumbar vertebra and rest of the tissue using a DL technique and analyzed the performance when our estimation network was fed either only the bone area or total area. The network was based on a deep residual convolutional neural network (CNN) (He et al., 2015) for visual tasks, and its capability was evaluated by Pearson’s correlation coefficient between BMD estimate and DXA reference.

In addition, we interpreted the DL results using explainable AI (XAI) techniques. XAI is currently used in medical fields to clarify DL features to assist clinicians in understanding and trusting decisions (Amann et al., 2020; Singh et al., 2020). In particular, the gradient-weighted class activation map (Grad-CAM) (Selvaraju et al., 2017) is popular in medical imaging because of a heat map highlighting important areas for decision making in an image. However, the method is theoretically specialized in a binary or multiclass classification task, where the map emphasizes areas for a target class while dismissing them for others; thus, it is not suitable for our estimation task. In this regard, we developed two new attention maps called gradient-weighted regression activation map (Grad-RAM) and Grad-RAM by pixel (Grad-RAMP) by modifying the Grad-CAM concept to specifically identify the local anatomical regions that have an impact on BMD prediction in every CT image.

## 2 Materials and methods

**FIGURE 1:**
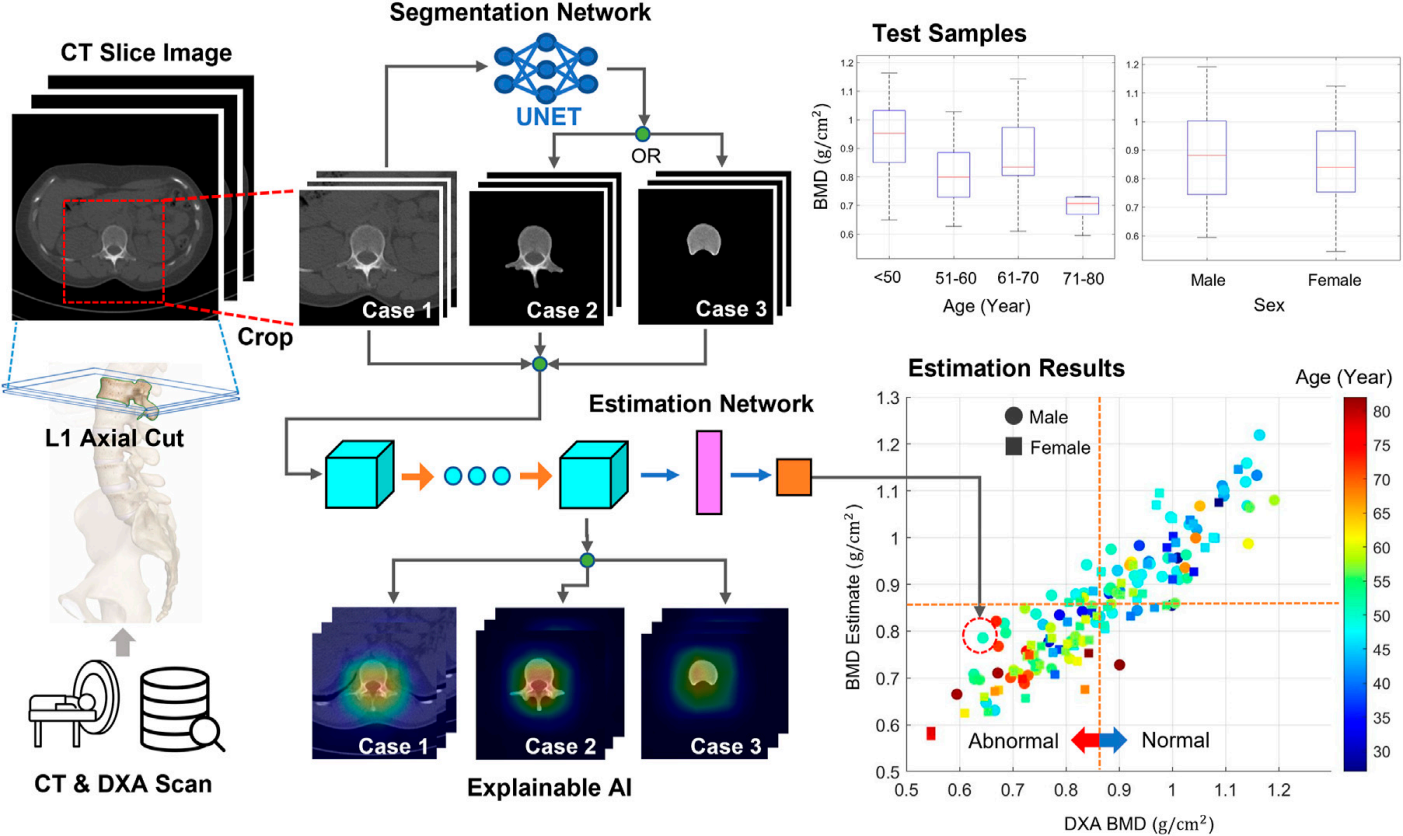
Workflow of this study. From the CT axial images, two image sets were generated. The first set included images cropped around lumbar vertebrae from original images. The second set included images highlighting only the bone body using a segmentation technique. The neural network was trained and tested using either image set for predicting BMDs. DXA BMDs were used as references for supervised learning. Bone diseases, such as osteoporosis and osteopenia, were screened using a T-score converted from every BMD. In addition, the attention map was obtained using XAI techniques to examine the important regions for the BMD prediction.

Figure 1 shows this workflow. Every full-size CT slice image was cropped to reduce the burden of the DL training. The DL network was trained and tested using cropped or bone-segmented images for BMD prediction. For the supervised learning scheme, DXA BMD values were used as references. The process was repeated by replacing the images with bone-segmented images, and the change in the test results was analyzed. Here, the bone area of interest was extracted from every cropped image using a DL network specialized in a segmentation task. Using the T-score obtained from a BMD estimate, every test sample was classified as either normal or abnormal. In addition, the local area critical for BMD prediction in every image was monitored using XAI techniques.

### 2.1 Data acquisition and equipment

From 6 March 2013, to 11 August 2020, the orthopedic team at Busan Medical Center collected 981^−CT^ volumes from 547 patients. Some patients provided multiple volumes, per person on different dates. This study was approved by the Institutional Review Board (P01-202009-21-009) at Busan Medical Center. The requirement for informed consent was waived considering the retrospective nature of the study and the use of anonymized clinical data. The collected data were under the condition that the date gap between CT and DXA scan was less than 1 month and the protocol was either chest, abdominal, or spine CT with a complete L1 axial cut. We determined a reference axial CT section that contained the maximum trabecular area of the L1 vertebral body from each CT volume. Then, we created a dataset in which every data sample consisted of the reference image and the corresponding DXA BMD. In addition, we added data samples to the dataset by selecting one or two of the nearest axial images from the reference for simple augmentation. The total number of samples were 2,696.

CT scanning procedures were performed on a Siemens (SOMATOM 128, Definition AS+) scanner (Siemens Healthcare, Forchheim, Germany) with a single-energy CT protocol, 120 kVp, 247 mA, dose modulation .6-mm collimation, effective pitch of .8, and B60 (sharp) reconstruction kernel. The reconstructed slice thickness for chest CT was set at 5.0 mm, 3.0 mm for lumbar spine CT, and 3.0 mm for abdomen and pelvis CT, with slice increments of 5.0 and 3.0 mm.

DXA measurements were performed during routine clinical examination at Busan Medical Center using a standard DXA device with a standard protocol (GE Lunar Prodigy, GE Healthcare). Using vendor-specific software, DXA images were automatically analyzed and reports were generated (Physicians Report Writer DX, Hologic, Discovery Wi, United States).

### 2.2 L1 segmentation

**FIGURE 2:**
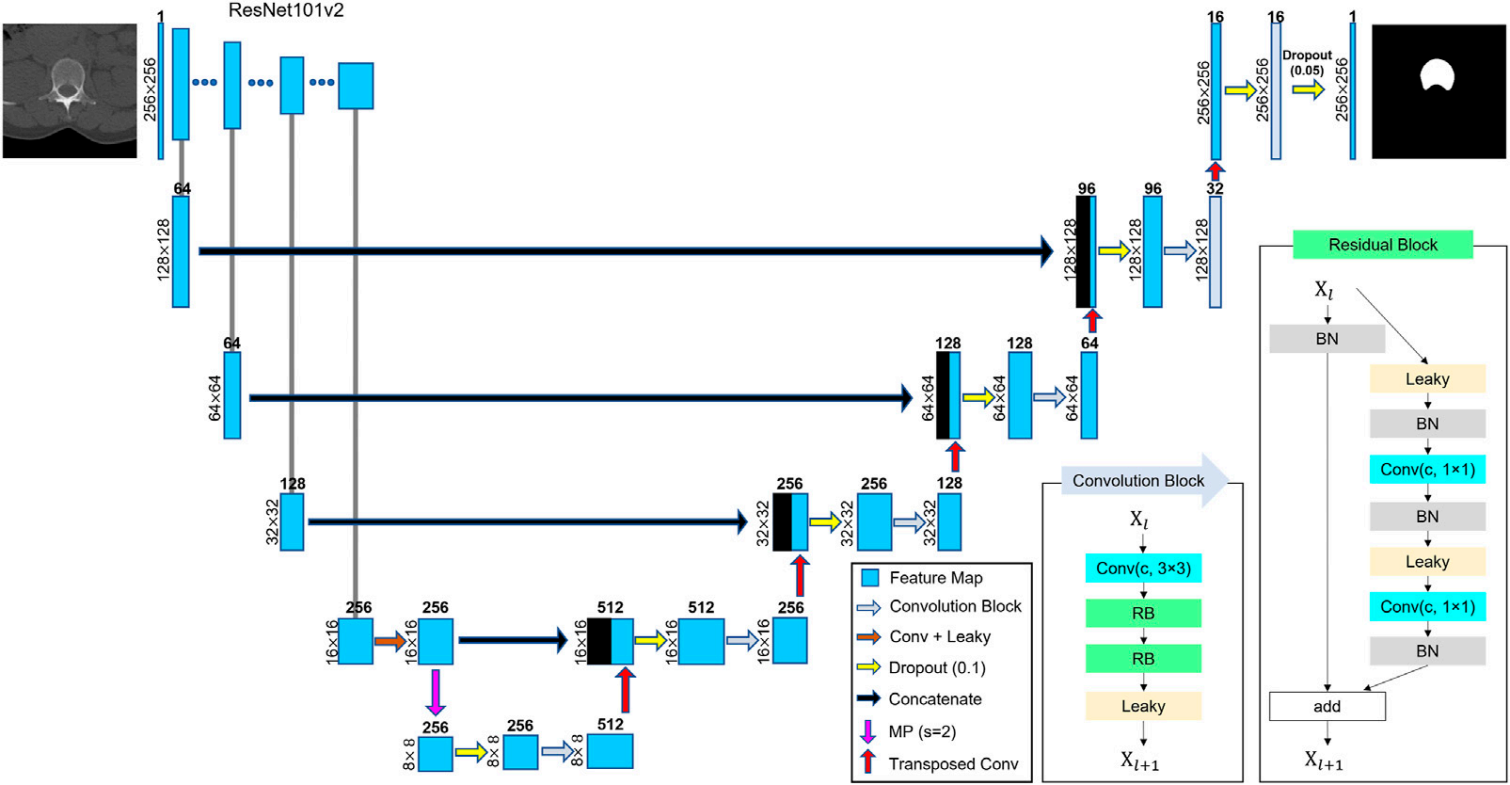
Schematic of U-Net structure. ResNet-101v2 was used as the backbone network. The number on the left of every box represents the size of image or feature map. The number above the box denotes the number of feature maps (channels). A dropout was applied before every convolutional block for generalization. Arrows represent the operators. The meanings of the abbreviations are as follows: Conv, convolutional layer; RB, residual block; Leaky, leaky ReLU ( α = .1); BN, batch normalization; MP, max-pooling layer; s, strides, and c, channel of convolutional layer.

Segmentation of either L1 entire vertebrae or vertebral body was performed using DL techniques. U-Net has been widely used in the field of semantic segmentation because of its effective feature extraction on multiple scales (Ronneberger et al., 2015; Alom et al., 2018; Diakogiannis et al., 2020). We adopted residual U-Net using ResNet-101v2 (He et al., 2015) as a backbone network, as shown in Figure 2, because ResNet-based models have been reported to enhance generalization in various tasks (Zhang et al., 2020). The network consisted of a residual block for encoding and multiple convolution blocks for decoding. Every convolution block was activated by a leaky rectified linear unit (leaky ReLU, α = .1) (Maas et al., 2013) function. The network included down and upsampling operators for multidimensional attention. The output layer was activated using a sigmoid function to determine whether every pixel corresponds to the target. After we randomly selected 204^−CT^ images from the training samples and labeled them to create L1 binary mask images, we trained the network using the CT and mask images. During training, the number of epochs was 500, batch size was eight, and optimizer was Adam. The loss function $f_{loss}$ was a dice coefficient (Dice, 1945) loss expressed as $f_{loss}=-\left(2\times \left|P\cap G\right|\right)/\left(\left|P\right|+\left|G\right|\right)$ where P and G denote the sets of positives (pixels) of prediction and ground truth, respectively, and $\left|\cdot \right|$ denotes the cardinality. The final trainable parameter values were selected when they resulted in the lowest loss. Owing to the U-Net model, we could automatically segment the total L1 bone or L1 vertebral body from the other CT images, as shown in Figures 6–8.

### 2.3 BMD prediction

**FIGURE 3:**
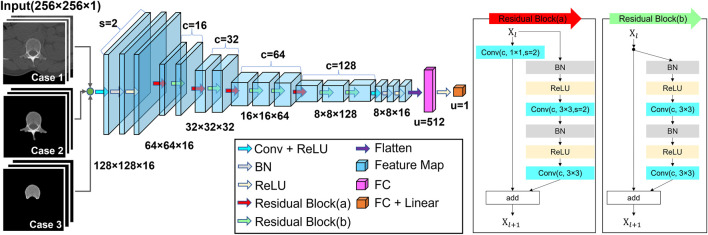
Schematic of the residual CNN structure. Inputs are either cropped images or bone-segmented images, and outputs are BMD estimates. When the residual block reduces the size of the feature map, the self-replicating stream has down-sampling using the 1 × 1 convolution with a stride of two. The meanings of the abbreviations are as follows: Conv, convolutional layer; BN, batch normalization; s, strides; c, channel of convolutional layer, and u, number of units.

**FIGURE 4:**
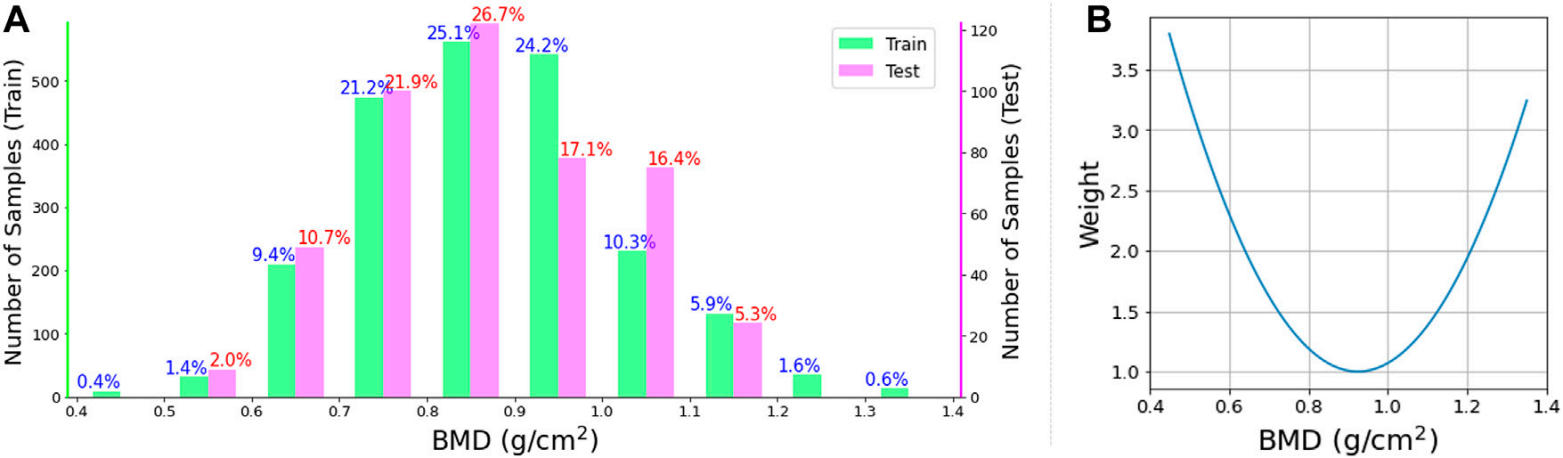
**(A)** Histogram of BMD. The left and right vertical axes denote the numbers (frequencies) of training samples and test samples, respectively. The horizontal axis denotes BMD and the bin width is .1. The number above every bar represents the percentage ratio. **(B)** Weight w used for the loss function in Eq. 1. The weight curve over BMD is associated with the inverse of the curve fitting to the distribution of training samples in the histogram.

A residual CNN model was developed for BMD predictions, as shown in Figure 3. The DL network consists of 22 convolutional layers and two fully connected layers. The interim 20 convolutional layers were placed in 10 residual blocks. The sizes of the feature maps gradually decreased in the feedforward direction by convolution with a stride of two. After every convolution layer, batch normalization was applied to stabilize learning. The activation function for each layer was a rectified linear unit (ReLU) (Nair and Hinton, 2010) function. Only the output layer was activated using a linear function. In the dataset, 2,239^−CT^ images (454 patients) were used to train the network, and 457^−CT^ images (93 patients) were used for the final test. The patients used for training were independent of those used for the test. Patient characteristics for training and test are summarized in Table 1. For robust training, traditional augmentation techniques were conducted by rotating images within 5°, shifting them within 10%, and horizontally flipping them. The experimental model was trained using either cropped CT images (Case 1), entire-vertebrae-masked images (Case 2) or vertebral-body-masked images (Case 3). The model was trained by minimizing the errors between the predicted values and DXA BMD (reference) values. Figure 4 shows a histogram of the reference BMD, where the bin size is .1. Because the distribution was not even, we used weighted Huber loss (Mangasarian and Musicant, 2000; Huber, 2011) ( δ=0.1 ) as the error metric. Let the histogram (frequency number) be $z\left(k_{y}\right)$ where $k_{y}$ denotes the bin index for the BMD y . We fitted the histogram curve to a simple quadratic curve, inverted and normalized the curve, and applied it to the error metric as follows:

$$e=\left\{\begin{array}{c} 0.5\times w\times {\left({\hat{y}}_{i}-y_{i}\right)}^{2},\;\;if\;\left|{\hat{y}}_{i}-{\;y}_{i}\right|\leq \delta \\ \delta \times w\times \left(\left|{\hat{y}}_{i}-y_{i}\right|-0.5\times \delta \right),\;\;\;otherwise \end{array}\right.,\;w=f\left(\frac{1}{z\left(k_{y_{i}}\right)}\right)$$ (1)

where w denotes the weight; ${\hat{y}}_{i}$ and $y_{i}$ denote the predicted and reference values for i th sample, respectively; and f denotes the normalization function with a minimum weight of 1.0. For updating, the epoch number, batch size, and optimizer were set as 150, 15, and Adam, respectively. During learning, 20% of the samples were randomly selected as a validation data set and the remaining samples were used as a training data set. This was repeated 10 times as cross-validation. During the tests, 10 consequently trained models independently predicted the BMD values for every test sample, and their average was used for the final BMD prediction as an ensemble technique. Pearson correlation coefficient and MAPE were computed to quantitatively measure the similarity between the predicted and reference BMDs using test samples.

**TABLE 1:** Patient characteristics of training and test dataset. Each row means number of male/female patients, mean age and standard deviation, mean time interval between CT and DXA scans and standard deviation, respectively.

	Training dataset	Test dataset
Men/women (*n*)	215/239	51/42
Mean age (years)	53.0 ± 10.4	52.1 ± 11.1
Mean time interval between the CT and DXA scan (days)	.5 ± 3.0	.5 ± 3.1

### 2.4 Disease classification

To diagnose the presence of bone diseases, we converted each BMD estimate into a T-score value: T-score = (BMD − $\mu_{ref}$ )/ $\sigma_{ref}$ (Frost et al., 2000) where the mean $\mu_{ref}$ and standard deviation $\sigma_{ref}$ of the population were .9747 and .1185, respectively. If the T-score was less than −1.0, we judged that the image sample was abnormal (osteoporosis or osteopenia); otherwise, the sample was normal. After classifying all samples, we obtained diagnostic results, such as accuracy, precision, specificity, sensitivity, and F1 score.

### 2.5 XAI analysis—Grad-RAM

Grad-CAM (Selvaraju et al., 2017) is a well-known XAI technique used to investigate the attention of a DL network toward an image. We modified Grad-CAM for this study because it is specialized in a classifier, whereas our network is an estimator (regressor). The localization map was obtained as follows:

$$L_{Grad-RAM}\left(i,j\right)=\left|\underset{k}{\sum }\alpha_{k}A_{k}\left(i,j\right)\right|,\;\alpha_{k}=\frac{1}{Z}\underset{i}{\sum }\underset{j}{\sum }\frac{\partial y}{\partial A_{k}\left(i,j\right)}$$ (2)

where k denotes the channel index of the convolutional layer, $A_{k}\left(i,j\right)$ denotes the k th feature map, $\alpha_{k}$ denotes the k th weight, and Z denotes the number of pixels in the feature map. Similar to Grad-CAM, the weight was obtained by averaging the gradients of the estimate y with respect to the feature map $A_{k}\left(i,j\right)$ of the last convolution layer. Then, the localization map was obtained using the absolute value of the linear combination of the feature maps with the weights. The method converted the ReLU operator in Grad-CAM to an absolute operator because it considered the features that have a significant impact on the estimate, regardless of the direction (sign) of the gradient. This method was named as “Grad-RAM” in this study. We obtained ten localization maps from ten predictive models for every sample and averaged them to obtain one final heat map. The map was superimposed onto the original CT image to visualize local attention.

### 2.6 XAI analysis—Grad-RAMP

We developed an additional technique named as “Grad-RAMP” by modifying the Grad-RAM. As shown in Eq. 3, Grad-RAM assigns one weight to all the pixels of every feature map during the linear combination. To enhance the localization of the gradient with respect to every pixel $\left(i,j\right)$ , we multiplied each gradient with the corresponding pixel as follows:

$$L_{Grad-RAMP}\left(i,j\right)=\left|\underset{k}{\sum }g_{k}⊙A_{k}\left(i,j\right)\right|,\;g_{k}=\frac{\partial y}{\partial A_{k}\left(i,j\right)}$$ (3)

where ⊙ denotes the Hadamard product operator. In addition, we obtained ten heat maps using this technique for every sample, averaged them, and superimposed a final map onto the corresponding CT image.

## 3 Results

### 3.1 BMD prediction

**FIGURE 5:**
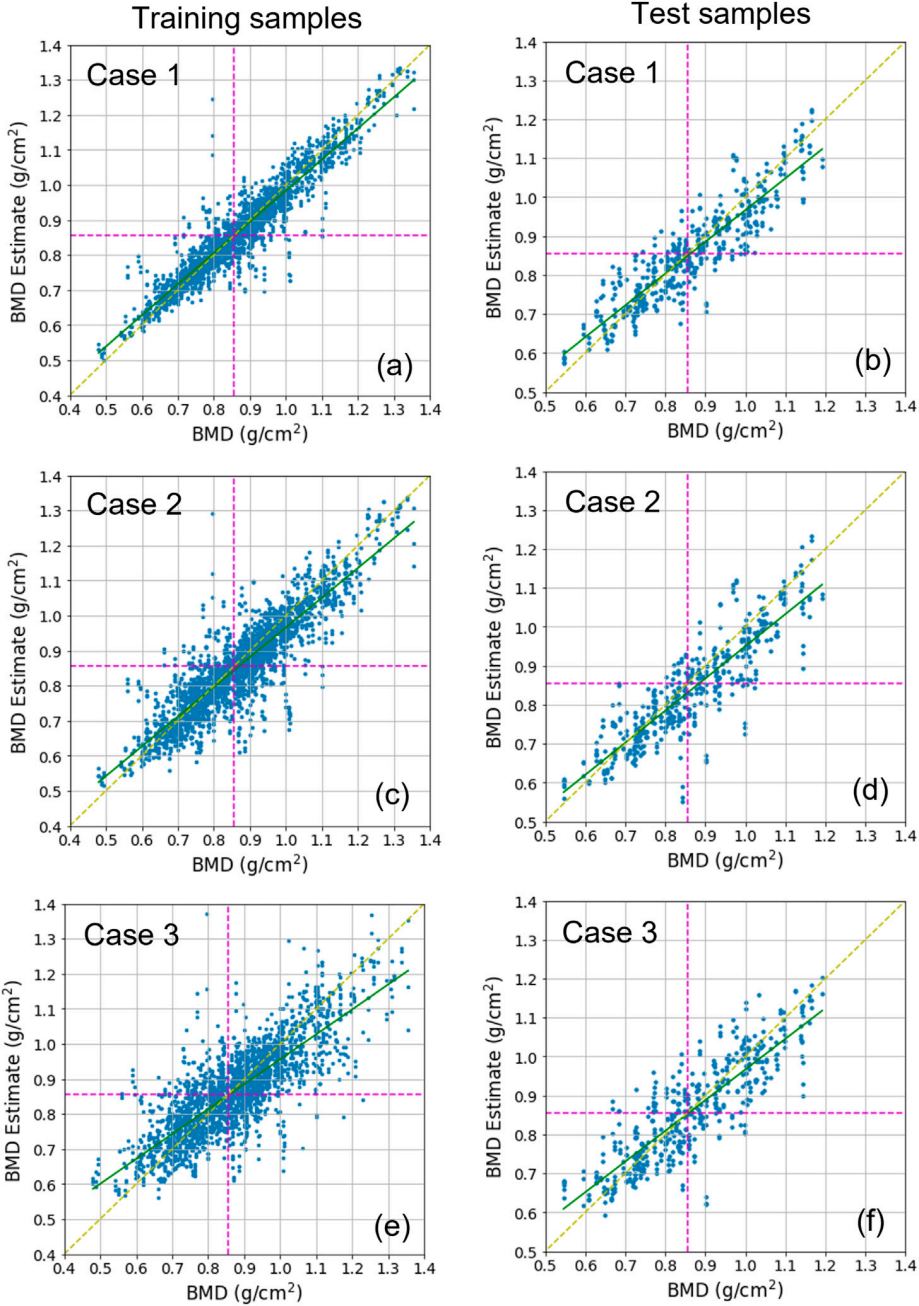
Scatter plots displaying BMD estimates over references. **(A,C,E)** show the training results, and **(B,D,F)** show the test results. **(A,B)** show Case 1 results, **(C,D)** show Case 2 results, and **(E,F)** show Case 3 results. In every plot, the green line is the line of the best fit for samples. The yellow dotted diagonal line denotes the ideal reference line for checking the skewness of the fitted line. The dotted horizontal and vertical line denotes the decision boundary for classifying bone diseases. The boundary value corresponds to .856 $g/cm^{2}$ as a BMD and −1.0 as a T-score.

Three cases were tested. Case 1 used datasets including cropped images. Case 2 and Case 3 used datasets including images segmented for overall L1 vertebra areas and only L1 vertebral bodies, respectively (Figure 1). For each case, the DL network for the estimation task used 2,239 training samples and 457 test samples. Figure 5 shows the BMD estimates over the references, using scatter plots. In every plot, the yellow dotted line indicates an ideal reference line and green line indicates a fitted line by the scatterers in a least-squares sense. The estimation was almost unbiased in the entire BMD range because the fitted line was very close to the ideal line for all cases. In addition, learning avoided overfitting problems because both the training and test results looked similar. Table 2 lists the quantitative results of the test samples. The correlation coefficients between the estimates and references for Case 1, Case 2, and Case 3 were .905, .878, and .853, respectively. The mean absolute percentage error (MAPE) for Case 1, Case 2, and Case 3 were 5.66, 6.81, and 6.90, respectively.

**TABLE 2:** Estimation test results and diagnostic scores for Case 1, Case 2, and Case 3. The estimation performance metric includes correlation coefficients and mean absolute percentage error (MAPE). The diagnostic performance metric includes accuracy, precision, specificity, sensitivity, and F1 score. Here, TP and FN denote true positive and false negative, respectively, where positive and negative denote abnormal and normal, respectively. All indicators are rounded to the third decimal place.

	Case1	Case 2	Case 3
Correlation coefficient	.905 (*p* < .001)	.878 (*p* < .001)	.853 (*p* < .001)
MAPE (%)	5.66	6.81	6.90
Accuracy	.862 (394/457)	.864 (395/457)	.825 (377/457)
Precision [TP/(TP + FP)]	.842 (208/247)	.822 (217/264)	.833 (190/228)
Specificity [TN/(FP + TN)]	.827 (186/225)	.791 (178/225)	.831 (187/225)
Sensitivity [TP/(TP + FN)]	.897 (208/232)	.935 (217/232)	.819 (190/232)
F1 score [(2 × precision × sensitivity)/(precision + sensitivity)]	.868	.875	.826

### 3.2 Bone disease classification

According to WHO standards, over 50-year-old men or postmenopausal women are diagnosed with osteopenia and osteoporosis if the BMD T-scores are less than −1.0 and −2.5, respectively (World Health Organization, 1994). It is considered as normal if the T-score is greater than −1.0. We converted the predicted and reference BMDs into T-scores and classified them using a T-score threshold of −1.0. This matches with approximately .856 as BMD, expressed by the vertical or horizontal dotted line in Figure 5. We then measured the accuracy, precision, specificity, sensitivity, and F1 score for each case. Table 2 lists the results. In all cases, the F1 score was greater than .8. In particular, Case 1 and Case 2 reached a F1 score of approximately .87. The table shows that overall, Case 1 and Case 2 were slightly better than Case 3. Some sample images in Case 1 are shown in Supplementary Figure S1 along with the prediction results.

### 3.3 XAI analysis

**FIGURE 6:**
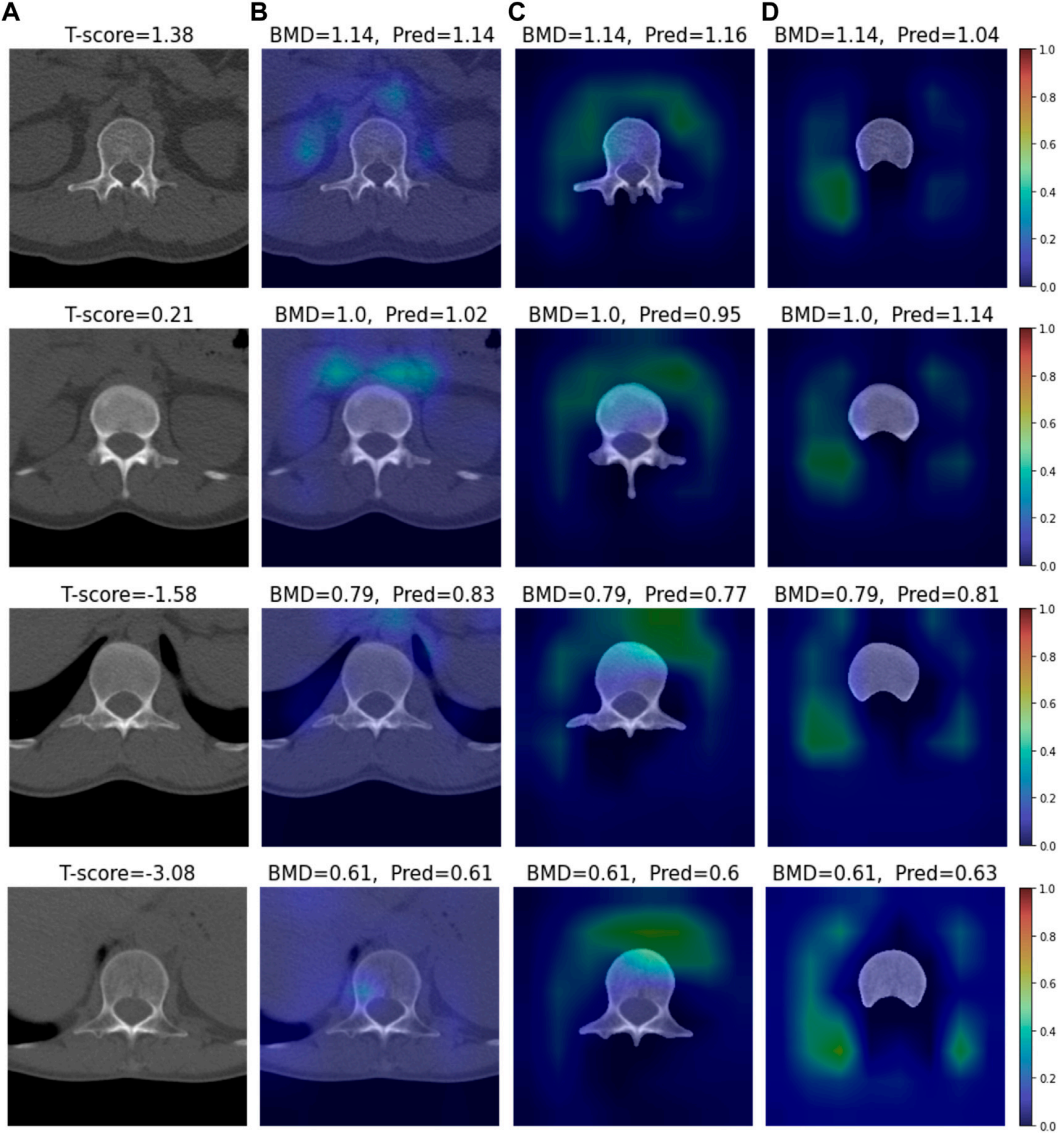
Visual XAI results (4 test samples) using Grad-CAM. The results of every sample are represented by every row. Every row, **(A)** represents the cropped CT image. A T-score value is placed at the top of every image. **(B–D)** display the heat map superimposed on the cropped image or bone-segmented image. **(B–D)** show Grad-RAMP results in Case 1, Case 2 and Case 3, respectively. “BMD” and “Pred” placed at the top of every image denote the reference and predicted values, respectively.

**FIGURE 7:**
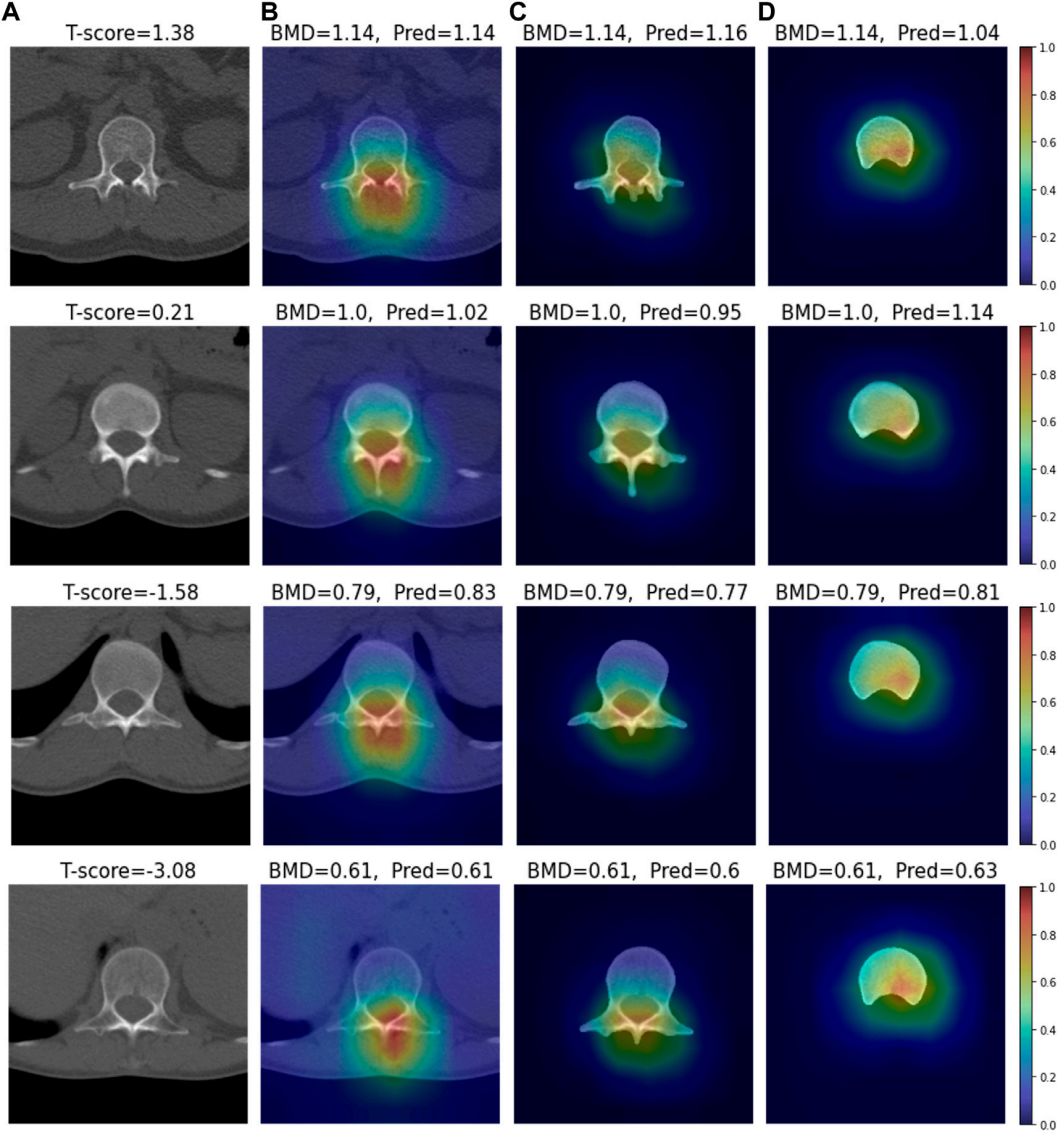
Visual XAI results (4 test samples) using Grad-RAM. The results of every sample are represented by every row. Every row, **(A)** represents the cropped CT image. A T-score value is placed at the top of every image. **(B–D)** display the heat map superimposed on the cropped image or bone-segmented image. **(B–D)** show Grad-RAMP results in Case 1, Case 2 and Case 3, respectively. “BMD” and “Pred” placed at the top of every image denote the reference and predicted values, respectively.

**FIGURE 8:**
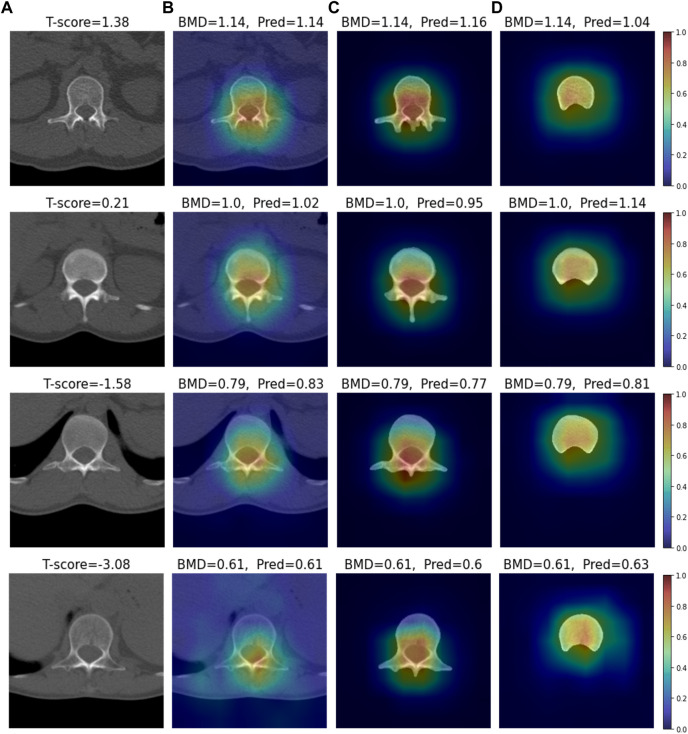
Visual XAI results (4 test samples) using Grad-RAMP. The results of every sample are represented by every row. Every row, **(A)** represents the cropped CT image. A T-score value is placed at the top of every image. **(B–D)** display the heat map superimposed on the cropped image or bone-segmented image. **(B–D)** show Grad-RAMP results in Case 1, Case 2 and Case 3, respectively. “BMD” and “Pred” placed at the top of every image denote the reference and predicted values, respectively.

We conducted visual explainable methods using Grad-RAM and Grad-RAMP. The purpose of this study was to investigate the local region in which our DL model critically estimated BMD. A standard technique, Grad-CAM, was not appropriate for this task, as shown in Figure 6 and Supplementary Figures S2, S3; thus, we devised new maps. Figures 7, 8 show the heat maps superimposed on the cropped image or bone-segmented images using Grad-RAM and Grad-RAMP, respectively. Four samples were randomly selected and vertically aligned over the T-score. In Case 1 and Case 2, the attention areas of the Grad-RAM were broadly located near the lumbar vertebra. Overall, the area focused on the foramen vertebra, spinosus process, and near tissues. Meanwhile, Grad-RAMP provided highlighted regions closer to the vertebral body and more centered at the vertebral foramen. In Case 3, the areas of Grad-RAM leaned toward one side of the vertebral body, whereas those of Grad-RAMP occupied the center of the body.

## 4 Discussion

This study investigated DL methods for predicting BMD using standard CT slice images. We generated three datasets and fed either set to the DL network during training, where the first set included CT images cropped around the L1 vertebra (Case 1), and second and third sets included their bone-segmented images (Case 2: total L1 bone, Case 3: only vertebral body). To guarantee robustness, we used large patient and image samples, and short scan intervals between CT and DXA. In either case, the trained network resulted in high diagnostic performance in the test with independent patient samples. As shown in Table 2, the correlation coefficient between the predicted and ground-truth values was greater than .85, and the mean absolute error was less than 7% in either case. In addition, the F1 score was greater than .82 in the disease classification task.

Overall, Case 1 and Case 2 outperformed Case 3, as shown in Table 2. This indicates that not only the vertebral body, but also other bone areas such as transverse process, spinous process and lamina contributed to BMD estimation. We noted that the reference for supervised learning was not real bone density at a narrow region, but measures obtained from DXA, whose scan spans large axial plans. Thus, it appears that our neural network extracted certain features from the rest areas as well as the bone body to access the reference in Case 1 and Case 2. A study showed that DXA BMD values depended on even the fat layer surrounding the bone (Colt et al., 2010). However, it appears that the soft tissue rarely affected the estimation in our experiments since Case 1 provided no noticeable prediction scores as compared with Case 2.

We obtained .90 as the maximum correlation coefficient (MCC) between estimates and references in Case 1 using 93 patients as test samples. It outperformed the previous study (Yasaka et al., 2020) achieving .85 as the MCC from 45 patients. Also, it was comparable with the state-of-the-art results in X-rays (Hsieh et al., 2021). The MCC numbers were .92 and .90 when the targets were hip and spine, respectively. We presume that mapping CT to DXA BMD is more challenging because an axial CT image is more estranged than an X-ray from DXA based on a coronal projection view. We expect that if the DL framework were designed to be fed by more than one CT slice (3D volume image) as an input, it would provide a better metric score.

During the network training, we applied a variable weight to the cost (loss) function, as shown in Eq. 1. Weight was inversely proportional to the frequency of the samples at the BMD. Thus, the network mitigated the sample imbalance problem by providing a more even error over the BMD, as shown in Supplementary Figure S4. To categorize the samples into disease or normal groups, we used −1.0 T-score and .856 BMD threshold for the binary diagnostic tests. They targeted osteopenia, a benign stage of osteoporosis. By adjusting the weight, the sensitivity can be increased at the expense of specificity, and *vice versa*. If a T-score less than −2.5 is considered as osteoporosis, our test set contained 49 osteoporosis samples. Because every image sample was predicted as a disease (estimate < −1.0), the sensitivity for the severe state was 1. This is the strength of the network designed as a BMD estimator (regressor) compared with a network that is directly classified.

The network for BMD estimation was based on the structure of residual CNN. The total number of trainable parameters were 1,614,273. When we trained the network using 2,239 samples, it took approximately 7.58 s per epoch. For the tests, we stored the model when the loss of validation data was the lowest during training. During the tests, the average computation time for predicting a BMD from one sample was approximately .006 s (2.58 s/457 samples). We used Python for the implementation and NVIDIA 2080Ti to run them on a computer.

In addition to the comparative analysis between different datasets, we used XAI techniques to investigate the local areas attended by the neural network to estimate the BMD. Currently, the requirement for XAI has steadily increased to support the reliability and transparency of DL models. Although many techniques for classification tasks have been studied and applied (Böhle et al., 2019; Panwar et al., 2020; Mondal et al., 2022), those for estimation (regression) tasks have seldom been proposed. Therefore, we developed Grad-RAM and Grad-RAMP by modifying Grad-CAM. In classification tasks, a network mainly uses either sigmoid or softmax as the output unit to output the probability (positive value). Thus, a feature map involving high positive gradients of a target class tends to increase the probability of the class. Grad-CAM has a ReLU function at the end of the process. Unlike Grad-CAM, Grad-RAM even uses negative gradients by replacing ReLU with an absolute function, as shown in Eq. 2. In the estimation tasks, a feature map involving high gradients, regardless of their signs, is likely to affect the prediction of a continuous output number.

In addition to this modification, Grad-RAMP takes advantage of gradient localization. The method does not globally weight the feature map but locally (pixel-wise) weights it using gradients, as shown in Eq. 3. This is in agreement with the concept of the saliency map technique (Simonyan et al., 2014), that a pixel with a higher gradient has a higher impact on the final decision. As shown in Supplementary Figures S2, S3, Grad-RAM and Grad-RAMP yielded more reasonable results than Grad-CAM in a simple estimation task. The task goal was estimating the average of pixel values in a rectangle around other disturbance figures in each image sample. In most samples, our new methods successfully highlighted the rectangle area in contrast to Grad-CAM.

As shown in Figures 7, 8, the attention areas obtained using both techniques span the overall vertebra in the transverse plane. The hot regions seldomly learn toward the vertebral body in Case 1 and Case 2. This agrees with the assumption in the comparative study that the network also pays attention to other bone parts. One limitation is that the resolution of the maps was not enough to identify that even vertebral foramen contributed to predict BMD. It appears that Grad-RAMP provided slightly more reasonable results than Grad-RAM in the tests because the attention area was near the centers of the entire vertebrae and vertebral body in Case I (or Case 2) and Case 3, respectively.

Our ongoing research is to combine the Grad-RAMP with layer-wise relevance propagation (LRP) (Montavon et al., 2017; Dobrescu et al., 2019; Kohlbrenner et al., 2020) to enhance the map resolution. LRP can provide contribution scores of each pixel for the regression output by simply propagating the prediction backward through the network layers. It is reported that LRP algorithms provided inconsistent and noisy maps, but a more detailed explanation of what pixels are relevant to the output. If they are complementary and well-balanced, the new map can give a more distinct explanation for the prediction.

The limitation of this study is that the data were collected from a single medical institution. In our future study, we will collect more CT images and corresponding DXA BMD values from several machines and hospitals to reduce bias and increase robustness. An expectation from a subsequent study is that BMD can be estimated from the bone areas where DXA hardly scans. To cope with diverse bone shapes, we plan to develop a DL technique based on a conditional generative adversarial network (cGAN) (Isola et al., 2017) to create plausible bone shapes with the condition of a labeled density. If available, the final product will be a 3D map of BMD as dual-energy CT.

## 5 Conclusion

This study leveraged a DL architecture to provide accurate BMD from standard CT. Using this DL model, CT taken for diverse medical purposes can be used to screen latent patients at risk for osteoporosis without additional costs and radiation exposures. We used DXA BMDs as references for supervised learning because most medical professionals are still accustomed to T-scores from DXA for evaluating osteoporosis or osteopenia. We found that narrowing ROI from a total CT slide view to a bone body was inconducive for enhancing performances because DXA depends on the projection of the total area. Overall, our DL-based regressor using the large ROI can provide BMD, that is highly correlated to the DXA BMD (maximum correlation coefficient > .9). The proposed explainable method supported that the regressor rather paid attention to the area near the center of the entire vertebrae. We expect that the DL-based BMD prediction from CT would be practical in actual clinical practice if it is further proved by more CT samples from diverse medical institutions. In addition, we intend to use the proposed explainable framework for any estimation (regression) task in the medical field.

## Data Availability

The original contributions presented in the study are included in the article/Supplementary Material, further inquiries can be directed to the corresponding author.
