# Supplementary material for: Prediction of bone mineral density in CT using deep learning with explainability
Source: Front Physiol. 2023 Jan 10;13:1061911. doi: 10.3389/fphys.2022.1061911 (PMC9871249; doi:10.3389/fphys.2022.1061911)
Supplement: Supplementary file 1 [file Table1.DOCX]

Supplementary Material

## Supplementary Figures


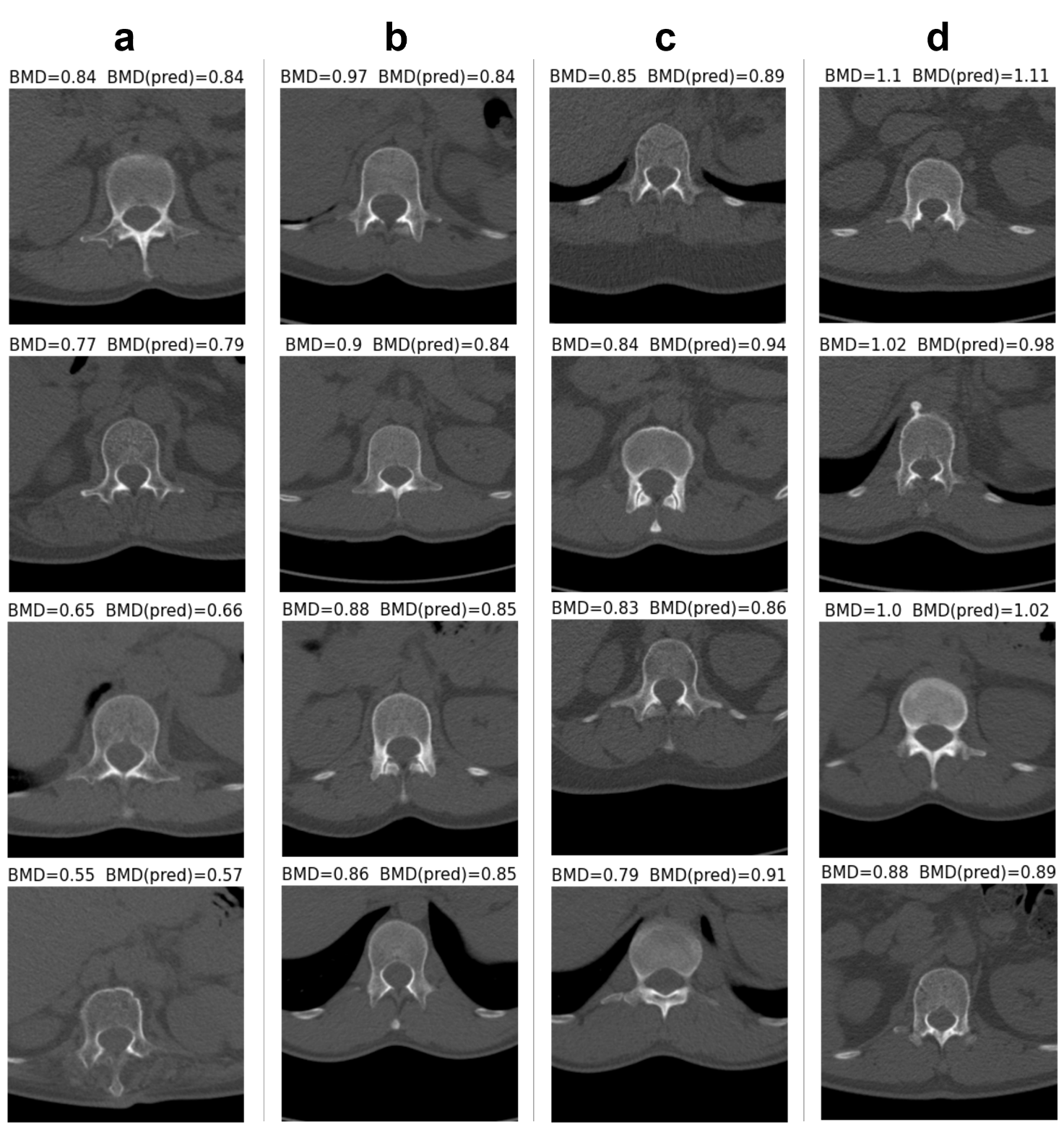


**Supplementary Figure 1.** CT sample images in the test set. They are used for testing the DL method for predicting BMD in Case 1. The words, “BMD” and “BMD(pred)” above every image indicate the reference BMD and estimate, respectively. Using the threshold of 0.856 (T-score=-1.0), the samples are binarily classified. When BMD is less than the threshold, the sample is abnormal (osteopenia), otherwise, it is normal. The classification outcome is either (**a**) true positive, (**b**) false positive, (**c**) false negative, or (**d**) true negative.


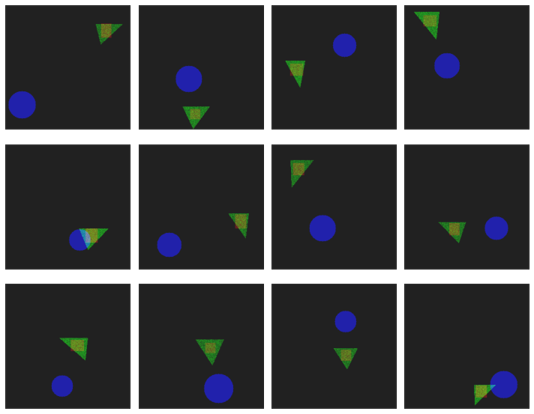


**Supplementary Figure 2.** Synthetic sample images for validating XAI techniques. Each image has circular, triangular, and rectangular figures. The size and position of each image are random within specific ranges. The triangle and relatively small rectangle mostly overlap. The pixel values in each figure are generated by random numbers from different Gaussian distributions. Given each sample image, the task goal is to find the mean of pixel values in the rectangle. Among a total of 1000 samples, all the above show only 12 samples. The network described in Fig. 3 is trained using the samples for estimating the mean value. The network provides around 0.97 as a correlation coefficient between estimate and ground-truth during tests with new samples. Here, it is checked which local area contributes the most to the estimation using XAI techniques.


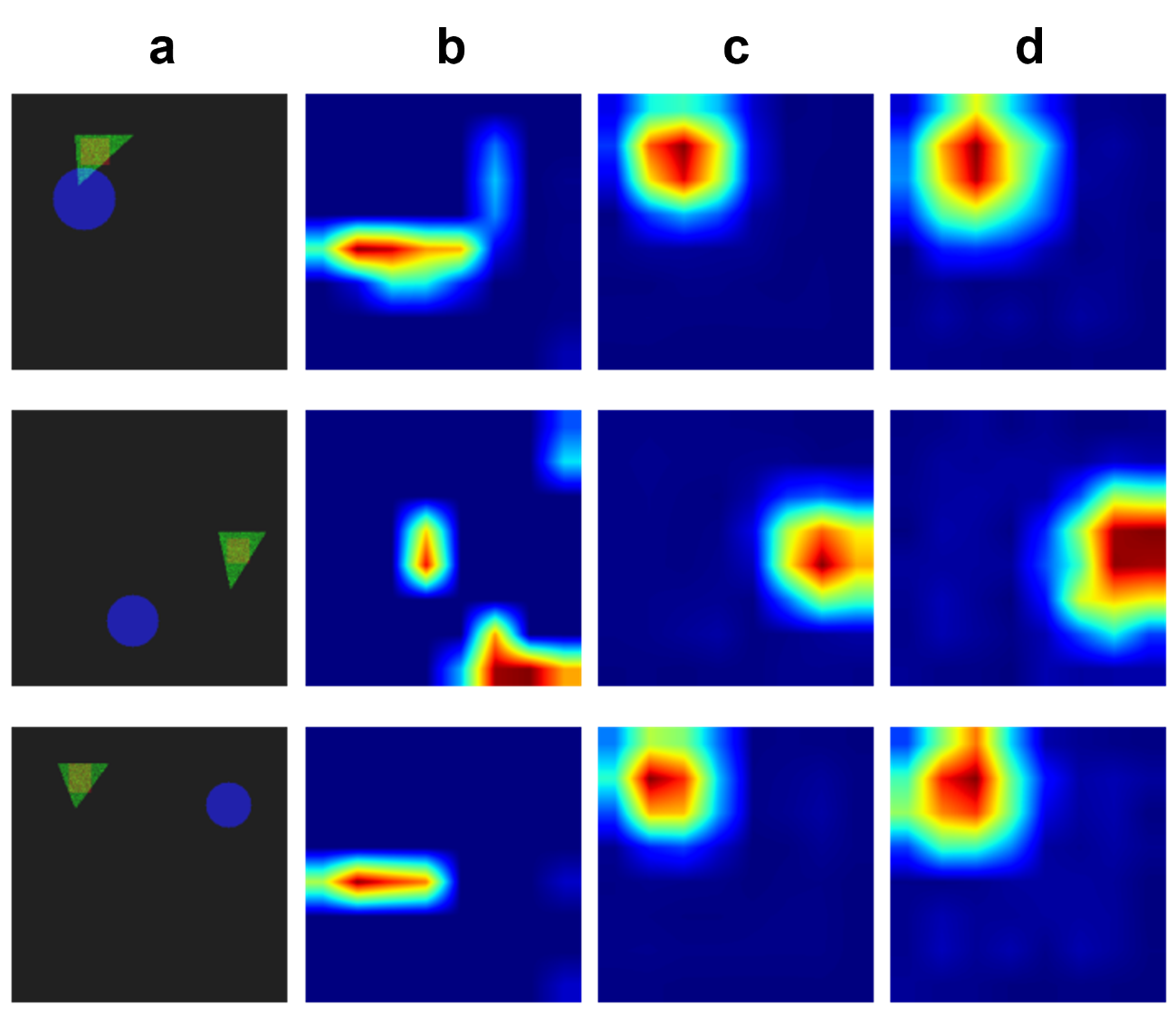


**Supplementary Figure 3.** XAI results using three synthetic samples. The experiment is described in Supplementary Fig. 2. (**a**) shows the test sample images. (**b**), (**c**) and (**d**) show the maps using Grad-CAM, Grad-RAM, and Grad-RAMP, respectively. In contrast to Grad-CAM, Grand-RAM and Grad-RAMP highlight the rectangular areas in each sample image.


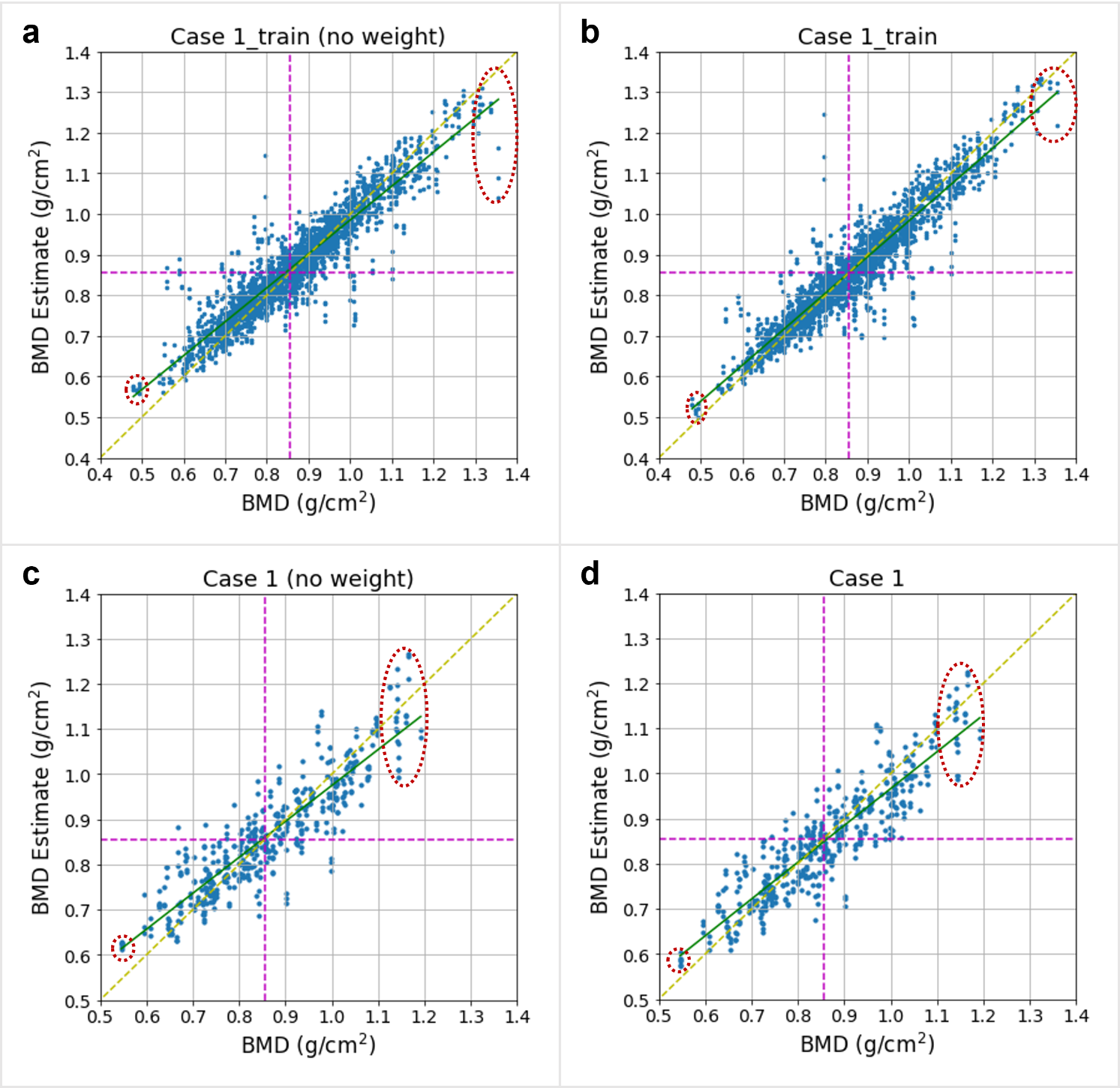


**Supplementary Figure 4.** Scatterplot of BMD estimate over reference BMD. As Figure 5, in every graph, the green line is the line of the best fit for samples, yellow dotted line is the ideal reference line, and purple dotted lines are the decision boundary for classifying the bone disease. (**a**) and (**b**) are estimates of training samples, and (**c**) and (**d**) are those of test samples. The dotted ellipses are for highlighting the effect of the weight in Huber loss (Eq. (1)). (**a**) and (**c**) are results of the loss without weighting while (**b**) and (**d**) are those with weighting.
